# Variability of response on prophylactic prasugrel for endovascular treatment of intracranial aneurysms: Clinical implications

**DOI:** 10.1371/journal.pone.0287190

**Published:** 2023-06-23

**Authors:** Noah Hong, Seung Bin Kim, Hee-Jin Yang, Young-Je Son

**Affiliations:** 1 Department of Neurosurgery, Seoul National University-Seoul Metropolitan Government Boramae Medical Center, Seoul, Republic of Korea; 2 Department of Critical Care Medicine, Seoul National University-Seoul Metropolitan Government Boramae Medical Center, Seoul, Republic of Korea; RKH Klinikum Ludwigsburg, GERMANY

## Abstract

**Background and purpose:**

Prophylactic prasugrel for endovascular treatment of intracranial aneurysms has been introduced and increased, but HTPR (high on-treatment platelet reactivity) or LTPR (low on-treatment platelet reactivity) of prasugrel is not uncommon in clinical circumstances. To investigate the predisposing factors of HTPR and LTPR on prasugrel premedication in the neurointerventional field and to determine its clinical implications.

**Materials and methods:**

Between February 2016 and December 2020, 191 patients treated with coil embolization using prophylactic prasugrel in 234 intracranial aneurysms were the final candidates for this study. Patient and aneurysm characteristics, clinical status, and laboratory study values were carefully reviewed retrospectively. We performed risk factor analyses for HTPR and LTPR on prasugrel.

**Results:**

Ultimately, 20 patients (10.5%) had HTPR, and 74 patients (38.7%) were categorized as having LTPR. In multivariable analyses, the factors related to HTPR were BMI (adjusted OR 1.21, 95% CI 1.04–1.41, p = 0.01), history of antithrombotics (adjusted OR 3.79, 95% CI 1.39–10.34, p = 0.01), and hematocrit (adjusted OR 0.91, 95% CI 0.84–0.99, p = 0.03). Low BMI was the only risk factor for LTPR (adjusted OR 0.84, 95% CI 0.76–0.94, p = 0.001).

**Conclusion:**

In the neurointerventional field, high BMI and prior use of antithrombotic agents were related to HTPR, and low BMI was associated with LTPR on prophylactic prasugrel. High hematocrit levels decreased the risk of HTPR. When preparing endovascular treatment for intracranial aneurysms, attention to patients with these clinical features is required to address the possibility of ischemic or bleeding complications.

## Introduction

Thromboembolism is the one of serious complications of endovascular treatment for intracranial aneurysms [1], so prophylactic antiplatelet medication has been recommended [2, 3]. One of the representative antiplatelet drugs is clopidogrel, but its variability of effect according to diverse individual natures has been observed, especially genetic alteration [3, 4].

Prasugrel, a recently developed antiplatelet agent, has several advantages compared to clopidogrel, such as independence to genetic polymorphism and intestinal hydrolysis [5], and due to its superiority of clinical outcomes, it has been increasingly used in neurointerventional fields [6, 7].

However, despite those characteristics of prasugrel, LTPR (low on-treatment platelet reactivity) or HTPR (high on-treatment platelet reactivity) of prasugrel is not uncommon in clinical practice. A considerable number of studies have investigated the pharmacodynamics and pharmacokinetics of prasugrel as well as analyzed the response to prasugrel after administration and its affecting factors in the domain of coronary heart diseases [8–12], but only a few reports have been conducted in the neurointerventional field [13, 14].

We aimed to investigate the factors predisposing LTPR and HTPR of prasugrel used as premedication in the neurointerventional field and to determine its clinical implications.

## Materials and methods

### Patient selection

In endovascular treatment for intracranial aneurysms, prasugrel instead of clopidogrel has been used routinely as a premedication since February 2016 in our institution. Between February 2016 and December 2020, two hundred fourteen patients with 263 aneurysms were treated with coil embolization. Among them, the cases of repeated treatments or multiple aneurysms in different sessions (four patients with six aneurysms), ruptured aneurysm cases (two patients and two aneurysms), and cases with a lack of laboratory studies such as platelet reactivity assay or serum lipid profiles were excluded. Finally, 191 patients with 234 aneurysms were selected as the final candidates for this study. Medical records were reviewed for various patient parameters and the values of laboratory studies. Our institutional review board approved the study protocol (IRB No. 30-2021-134). The requirement to obtain written informed consent for study participation was waived. Data are publicly available upon reasonable request.

### Prasugrel premedication and endovascular procedure

Referring to previous research about low-dose prasugrel premedication in coil embolization for unruptured intracranial aneurysms [7, 14–16], all patients eligible for this study were routinely administered a 20 mg loading dose of prasugrel on the day before the endovascular procedure, and 5 mg of prasugrel was administered on the morning of the treatment. If stent implantation during the procedure was anticipated, we added a 300 mg loading dose of oral ASA (acetylsalicylic acid) at the time of prasugrel loading and maintained 100 mg of ASA with prasugrel on the day of the procedure.

In every case, intravenous unfractionated heparin was administered by bolus injections (3000 IU) just after insertion of the introducing sheath, supplemented by hourly doses (1000 IU) that were decided by activated clotting times.

Simple coiling by aneurysmal selection using a single microcatheter or multiple microcatheter techniques, and balloon or stent-assisted techniques were combined with various methods according to the physician’s decisions.

### Acquisition of blood samples and estimation for residual platelet reactivity

Whole blood was obtained immediately after taking a maintenance dose of prasugrel or prasugrel with ASA on the morning of the procedure. Residual platelet reactivity was measured using the VerifyNow P2Y12 assay (Accriva Diagnostics, San Diego, CA, USA), which provided the values of the BASE, the P2Y12 reaction unit (PRU), and percentage inhibition. BASE means baseline platelet reaction unit and PRU implies residual P2Y12 receptor activity after loading of antiplatelet agents. Percentage inhibition is calculated as follow: {(*BASE−PRU*)/*BASE*}×100. The lower PRU value and the higher percentage inhibition, the lower residual platelet reactivity, and vice versa.

### Clinical and radiological evaluation

Patient status was assessed using the modified Rankin scale (mRS) [17] at the time of admission, discharge, and 6 months after treatment. Periprocedural complications, including technical and procedure-related problems defined as thromboembolic events during the procedure or discovered ischemic insults clinically and radiologically, and any hemorrhagic problems were estimated to have a relationship with endovascular procedures and were carefully reviewed. We also screened angiographic features of treated aneurysms, such as the locations and devices used to assist the packing coils and protect parent arteries. Immediate angiographic results of the treatment were evaluated using the Raymond-Roy occlusion classification [18].

### Statistical analysis

Continuous variables are presented as mean ± standard deviation (SD) with range. Categorical variables are expressed as numbers and percentages of total cases. The Wilcoxon signed-rank test was used to compare neurological status over time. Risk factor analyses were performed by logistic regression to produce the odds ratio (OR) with a 95% confidence interval (CI) for the risk factor evaluation of HTPR and LTPR. HTPR and LTPR were defined as PRU values ≥ 208 and < 85 [19]. Variables with a p-value > 0.2 in univariate analysis and deemed fundamental factors, such as age or gender, were selected for multivariate analysis in a backward elimination. A p-value < 0.05 was considered statistically significant for all statistical tests. All statistical tests were performed using SPSS Statistics for Windows, version 27.0 (IBM Corp., Armonk, NY, USA).

## Results

### Basal characteristics

The baseline characteristics of 191 patients and 234 treated unruptured intracranial aneurysms are summarized in **Tables 1 and 2**. Endovascular treatment for multiple intracranial aneurysms via a single session was performed in 32 patients (16.6%) with 74 aneurysms (31.4%).

**Table 1:** Baseline characteristics of patients.

Characteristic	Treated patients
Number of patients	191
Sex, No. (%)	
Male	52 (27.2)
Female	139 (72.8)
Age in a year, mean ± SD (range)	64.7 ± 10.4 (36–83)
Body mass index, kg/m^2^, mean ± SD (range)	24.6 ± 3.2 (18.2–35.0)
Underlying diseases, No. (%)	
Hypertension	119 (62.3)
Diabetes mellitus	33 (17.3)
Hyperlipidemia	90 (47.1)
Previous stroke	33 (17.3)
Coronary heart disease	9 (4.7)
Arrhythmias	8 (4.2)
Current smoking, No. (%)	27 (14.1)
Alcohol consumption, No. (%)	66 (34.6)
Prior antithrombotic medications to treatment, No. (%)	61 (31.6)
With proton pump inhibitors, No. (%)	20 (10.5)
Laboratory tests, mean ± SD (range)	
LDL cholesterol, mg/dL	98.8 ± 31.6 (40–246)
HDL cholesterol, mg/dL	49.5 ± 15.9 (25–162)
Hematocrit, %	38.2 ± 5.3 (11.2–66.0)
Platelet count, ×10^3^/μL	245.9 ± 206.2 (78–2912)
PRU	115.5 ± 71.7 (1–392)
Percent inhibition, %	55.5 ± 26.7 (0–100)

LDL, low-density lipoprotein; HDL, high-density lipoprotein; PRU, P2Y12 reaction unit

**Table 2:** Baseline characteristics of treated aneurysms.

Characteristics	Treated aneurysms
Number of aneurysms	234
Previously treated aneurysms, No. (%)	10 (4.3)
Location, No. (%)	
Internal carotid artery	101 (43.2)
Paraclinoid	44
Posterior communicating artery	29
Dorsal ICA	8
Anterior choroidal artery	7
Cavernous	6
Other locations of distal ICA	4
ICA bifurcation	2
Ophthalmic	1
Anterior cerebral artery	63 (26.9)
Anterior communicating artery	52
A1	7
A2-3 junction	3
A2	1
Middle cerebral artery	48 (20.5)
MCA bifurcation	35
M2	7
M1	5
M2-3 junction	1
Posterior circulation	22 (9.4)
Basilar top	16
Vertebral artery	3
PICA	2
SCA	1
Procedure-assisted technique, No. (%)	
Multiple microcatheter	33 (14.0)
Stent	57 (24.4)
Balloon	9 (3.8)
Treatment result, No (%)	
Complete exclusion	39 (16.7)
Residual neck	132 (56.4)
Residual sac	63 (26.9)

ICA, internal carotid artery; MCA, middle cerebral artery; PICA, posterior inferior cerebellar artery; SCA, superior cerebellar artery.

### Clinical outcomes and periprocedural complications

There was no significant difference in mRS between the timing of admission and discharge. However, a comparison between admission and the time of 6 months after treatment showed a significant change (p < 0.001), with a lower mean mRS at 6 months post-procedure.

Periprocedural complications occurred in 17 patients (8.9%). Among these, 10 patients were symptomatic, and the other 7 patients had no signs or deficits. There were two mortality cases. One was the occurrence of intraprocedural rupture of the targeted aneurysm, and the other was retroperitoneal bleeding related to femoral puncture in the general ward after endovascular treatment, resulting in hypovolemic shock.

The detailed clinical outcomes according to the mRS and periprocedural complications are presented in **Tables 3 and 4**.

**Table 3:** Clinical outcomes evaluated by modified Rankin scale.

Modified Rankin Scale	Mean ± SD (range)	Median value (IQR)	P
At admission	0.5 ± 0.7 (0–3)	0 (1.0)	-
At discharge	0.5 ± 0.9 (0–6)	0 (1.0)	0.57*
6 months after treatment	0.3 ± 0.7 (0–5)	0 (0.0)	< 0.001*

IQR, interquartile range

*p-value compared to the state at admission

**Table 4:** Summary of periprocedural complications.

Patient No.	Treated aneurysm	Problem	Symptom	mRS		
				At admission	At discharge	
17	PCoA	Intraprocedural rupture	-	1		1
24	BAB	Infarction	Dysmetria	0		1
30	BAB	Perilesional edema	Hemiparesis	3		3
55	Paraclinoid ICA	Infarction	Motor aphasia	0		1
83	BAB	Closing device failure	-	1		1
94	M1	Intraprocedural rupture	-	0		6
96	MCAB	Infarction	Motor aphasia	2		2
98	M1	Distal branch occlusion	-	1		1
123	MCAB	Infarction	Altered mentality	2		2
124	Cavernous ICA	Oculomotor nerve palsy	Ptosis	1		2
130	PCoA	Retroperitoneal bleeding	Hypovolemic shock	0		0
134	Paraclinoid ICA	Coil migration	-	0		0
143	Dorsal ICA	Coil stretch	-	0		0
145	MCAB	Retroperitoneal bleeding	Hypovolemic shock	0		6
147	PCoA	Early detachment of the coil	-	1		1
172	Cavernous ICA	Infarction	Seizure	0		0
183	PCoA	Infarction	Arm weakness	0		2

mRS, modified Rankin scale; PCoA, posterior communicating artery; BAB, basilar artery bifurcation; ICA, internal carotid artery; MCAB, middle cerebral artery bifurcation

### Residual platelet reactivity and risk factors of both extremes

**Fig 1:**
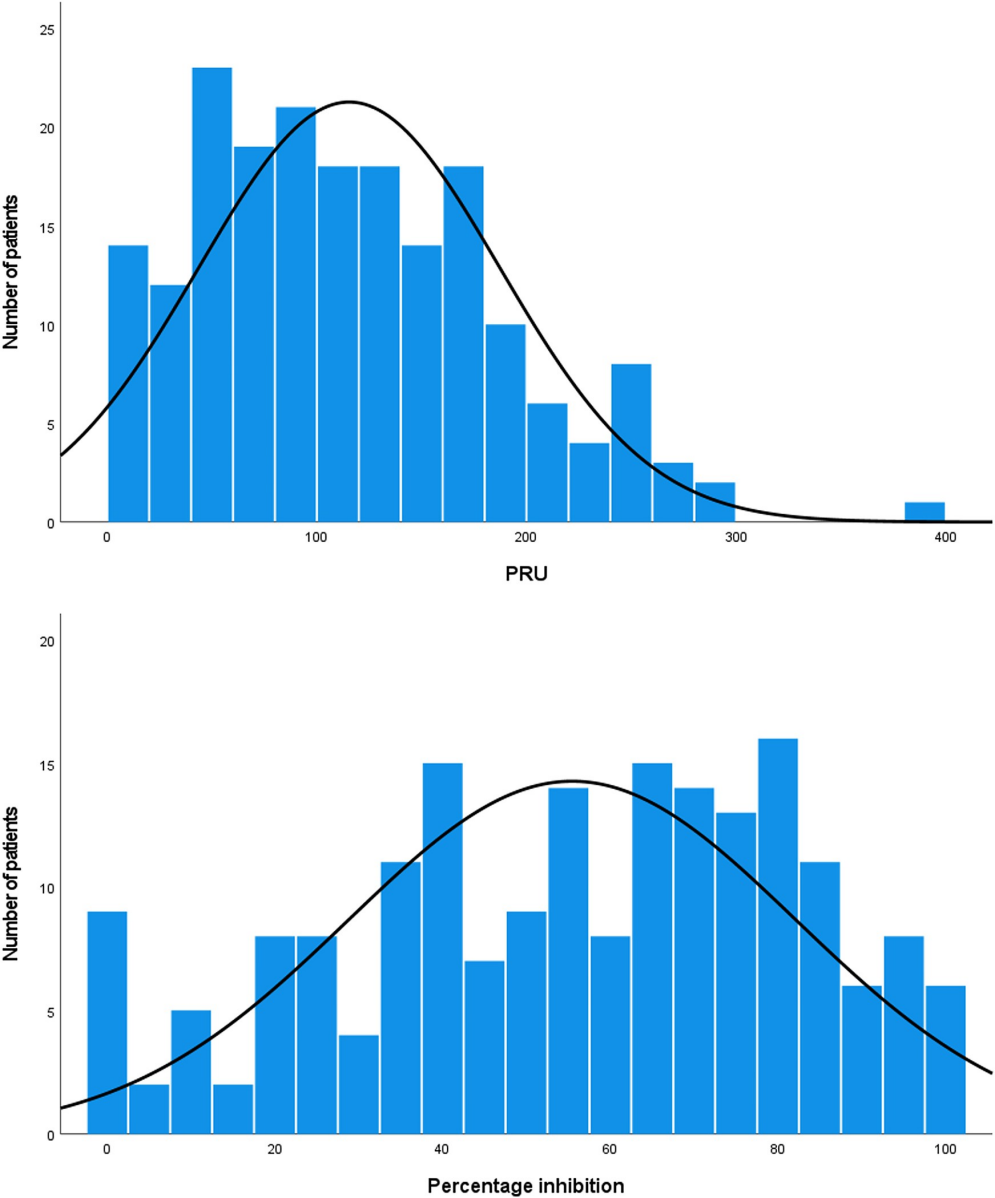
The histograms for distribution of P2Y12 reaction unit (PRU) and percent inhibition measured by VerifyNow P2Y12 assay.

**Fig 1** shows the distributions of PRU and percentage inhibition values derived from the VerifyNow assay. The mean PRU value was 115.5 ± 71.7 (range 1–392), and the median was 108. The mean percentage inhibition was 55.5% ± 26.7 (range 0–100%), and the median was 59%.

Among 191 individuals, 20 patients (10.5%) had HTPR associated with prasugrel, and 74 patients (38.7%) were categorized as having LTPR. **Tables 5 and 6** describe the results of the risk factor analysis. In multivariable analyses, the factors related to HTPR on prasugrel were BMI (crude OR 1.17, 95% CI 1.01–1.34, p = 0.03; adjusted OR 1.21, 95% CI 1.04–1.41, p = 0.01), previous use of antithrombotics (crude OR 2.96, 95% CI 1.16–7.58, p = 0.02; adjusted OR 3.79, 95% CI 1.39–10.34, p = 0.01), and hematocrit (crude OR 0.94, 95% CI 0.988–1.02, p = 0.13; adjusted OR 0.91, 95% CI 0.84–0.99, p = 0.03). Low BMI was the only risk factor for LTPR on prasugrel (crude OR 0.86, 95% CI 0.78–0.95, p = 0.004; adjusted OR 0.84, 95% CI 0.76–0.94, p = 0.001). Among the 61 patients with a prior history of antithrombotic agents, 56 (91.8%) had taken antiplatelet medications, and 40 individuals were chronic ASA users. Twelve patients were taking clopidogrel, two patients with cilostazol, and the other two patients were administered triflusal.

**Table 5:** Risk factor analysis of high on-treatment platelet reactivity (HTPR).

Variables	Univariate Analysis		Multivariate Analysis	
	OR (95% CI)	P	OR (95% CI)	P
Age	1.02 (0.97–1.06)	0.53		
Female over male	0.66 (0.25–1.77)	0.41		
BMI	1.17 (1.01–1.34)	0.03	1.21 (1.04–1.41)	0.01
Hypertension	1.93 (0.67–5.57)	0.22		
Diabetes mellitus	1.70 (0.57–5.06)	0.34		
Chronic kidney disease	0.00 (0.00-∞)	1.00		
Arrhythmia	0.00 (0.00-∞)	0.99		
Coronary heart disease	2.60 (0.50–13.49)	0.25		
Previous ischemic stroke	1.22 (0.38–3.93)	0.73		
Current smoking	1.08 (0.29–3.97)	0.91		
Alcohol consumption	0.60 (0.21–1.73)	0.35		
Drug history of antithrombotics	2.96 (1.16–7.58)	0.02	3.79 (1.39–10.34)	0.01
Drug history of proton-pump inhibitors	3.47 (1.12–10.87)	0.03		
Level of LDL cholesterol	1.00 (0.99–1.02)	0.89		
Level of HDL cholesterol	0.98 (0.94–1.01)	0.21		
Platelet count	0.99 (0.99–1.01)	0.65		
Hematocrit	0.94 (0.88–1.02)	0.13	0.91 (0.84–0.99)	0.03

BMI, body mass index; CI, confidence interval; high-density lipoprotein; LDL, low-density lipoprotein; OR, odds ratio

**Table 6:** Risk factor analysis of low on-treatment platelet reactivity (LTPR).

Variables	Univariate Analysis		Multivariate Analysis	
	OR (95% CI)	P	OR (95% CI)	P
Age	0.98 (0.95–1.01)	0.20		
Female over male	1.39 (0.71–2.72)	0.34		
BMI	0.86 (0.78–0.95)	0.004	0.84 (0.76–0.94)	0.001
Hypertension	0.87 (0.48–1.59)	0.65		
Diabetes mellitus	0.46 (0.19–1.08)	0.07		
Chronic kidney disease	0.00 (0.00-∞)	1.00		
Arrhythmia	0.97 (0.22–4.18)	0.97		
Coronary heart disease	1.31 (0.34–5.05)	0.70		
Previous ischemic stroke	1.06 (0.49–2.29)	0.88		
Current smoking	1.35 (0.59–3.08)	0.47		
Alcohol consumption	0.66 (0.35–1.23)	0.19		
Drug history of antithrombotics	0.64 (0.33–1.21)	0.17		
Drug history of proton-pump inhibitors	1.09 (0.42–2.80)	0.86		
Level of LDL cholesterol	1.004 (0.99–1.01)	0.43		
Level of HDL cholesterol	1.01 (0.99–1.03)	0.40		
Platelet count	1.003 (0.99–1.01)	0.12	1.004 (1.00–1.01)	0.05
Hematocrit	1.01 (0.96–1.07)	0.66		

BMI, body mass index; CI, confidence interval; high-density lipoprotein; LDL, low-density lipoprotein; OR, odds ratio

## Discussion

The rate of periprocedural symptomatic thromboembolism in coil embolization for intracranial aneurysms was reported as approximately 2.4% to 5.2% [20, 21], and this rate may have increased to include silent ischemia as high as 28% [22]. Prophylactic antiplatelet medications for endovascular procedures have been recommended for the prevention of thromboembolism with significant risk reduction [2, 3], particularly, in cases treated with multiple microcatheter techniques, this benefit was more evident [23].

Clopidogrel [2, 3, 24] and a combined regimen with ASA are generally accepted as standard prophylactic medications in coil embolization for unruptured intracranial aneurysms [2, 3, 23, 24]. Clopidogrel binds to P2Y12 adenosine diphosphate (ADP) receptors on platelets irreversibly and interrupts ADP-mediated activation of the glycoprotein IIb/IIIa complex [5], and this process requires a 2-step hepatic cytochrome P450 (CYP). The problem with clopidogrel is patients’ resistance to its antiplatelet effect, as it is known to have a higher risk of procedure-related thromboembolic complications in these patients, compared to patients who have no resistance to clopidogrel [24]. Genetic polymorphisms on the CYP2C19 allele have been accepted as the main cause of this resistance; one of the CYP isoenzymes produces low-of-function resulting in decreased activation of clopidogrel and antiplatelet effects. These genetic alterations were reported to be 14% to 55% in Asian populations of non-white and non-black ethnicity [5, 25].

Prasugrel is a 3^rd^ generation thienopyridine antiplatelet agent. It has unique differences in metabolic processes compared to clopidogrel, which is independent of hydrolysis by intestinal carboxylesterase 1. Prasugrel also has only a single CYP-dependent step and no influence by genetic polymorphism of CYP [5, 8]. Consequently, prasugrel has more effective inhibition of platelet aggregation, faster onset of action, stronger potency, and less response variability in individuals compared to clopidogrel. With these advantages, administration of prasugrel than clopidogrel showed better clinical outcomes in patients with coronary heart disease [26] and was introduced in neurointerventional procedures as a safe and effective premedication [7, 14]. According to Cho et al., low-dose prasugrel premedication showed good clinical outcomes and was more effective in reducing thromboembolic events than tailored clopidogrel-based prophylaxis for coil embolization of intracranial aneurysms [7].

Despite less variability of its antiplatelet effect, we recognized that HTPR or LTPR of prasugrel measured by P2Y12 assay were not absent in endovascular treatment of intracranial aneurysms; thus, we decided to investigate the predisposing factors related to responsiveness to prasugrel in the neurointerventional field.

We presented the predisposing factors of HTPR and LTPR on prasugrel, using loading dose as prophylactic antiplatelet medication in patients with intracranial aneurysms treated by endovascular procedures, and found that increased BMI was associated with a higher risk of HTPR and lower risk of LTPR, and history of antithrombotic drug use was related to a higher risk of HTPR, while increased hematocrit corresponded with a lower risk of HTPR.

BMI is known as a common factor influencing the pharmacokinetics of prasugrel in several studies [8, 27], especially in patients with a body weight < 60 kg [8]. Lower clearance of Pras-AM (active metabolites of prasugrel) in patients with low body weight results in overexposure to Pras-AM and causes pharmacokinetic alteration [8]. Cuisset et al. described that diabetes influences thienopyridine metabolism and affects the response to prasugrel, resulting in HTPR on prasugrel [28]. Other various factors such as sex, genetic variation of CYP enzymes, moderate hepatic function impairment, end-stage renal disease, proton pump inhibitors (PPI), and cigarette smoking act on the function of CYP subtype, and previous or concurrent use of clopidogrel, heparin, or warfarin were reported to not affect the residual platelet reactivity of prasugrel [12]. In the neurointerventional field, only a limited number of studies have mentioned factors affecting the response to prasugrel. Ha et al. reported a significantly low PRU value using the VerifyNow assay in patients with lower body weight under 60 kg, higher serum LDL cholesterol, cigarette smoking, and statin medication use [14], but those results showed only the tendency of low PRU value in groups with limited features and without multifactorial adjustment. Although our results did not show novelty, there is a distinguishing point in that our study is an investigation with a patient group belonging to the neurointerventional field. In prior large-scale and well-designed studies that investigated the efficacy and the factors related to prasugrel’s action within the field of coronary heart disease [29, 30], of course, the proportion of the patients with cardiovascular risk factors (hypertension, diabetes, hyperlipidemia, high BMI, and cigarette smoking) was much higher than the participants of our research. We cautiously suggest that this study is meant to know the factors that similarly affect prasugrel responsiveness in a more common population with unruptured aneurysms, which are not biased toward multiple cardiovascular predisposing factors.

It was unexpected that previous use of antithrombotic medications increases the risk of HTPR on prasugrel because we envisaged that the patients with long-term administration of antithrombotic agents had remaining antiplatelet or anticoagulation effects in their bodies and that this would decrease the residual platelet reactivity on prasugrel. However, these drug histories were diametrically predisposed to the risk of HTPR on prasugrel. We explored the details of antithrombotic medications that the patient used before endovascular treatment and found that a large proportion of the drugs (91.8%) were antiplatelet agents, and among them, most of the medications were ASA. A previous study reported an association between chronic use of ASA and slower response to prasugrel loading [9]. The authors of that study explained that lower platelet inhibition with prasugrel in long-standing ASA users with stable coronary artery disease compared to the healthy population, and worse outcomes of patients on ASA therapy with the acute coronary syndrome, seemed to describe the relationship between prasugrel and the characteristics of patients taking ASA in the long term, rather than a direct connection between chronic ASA use itself and prasugrel. Although we did not know the exact reason for taking ASA before treatment for intracranial aneurysms in our patients, among 40 individuals taking ASA chronically, only 6 patients had coronary heart disease. Hypertension (33 patients, 82.5%) and hyperlipidemia (25 patients, 62.5%) were the characteristics that account for the majority of chronic ASA users, but these were insignificant factors related to prasugrel responsiveness. We could not find anything in these patients that might be related to HTPR on prasugrel, and the ambiguous relationship between prior use of antithrombotics and HTPR on prasugrel remained unsolved.

We could not find any studies on hematocrit and HTPR on prasugrel; instead, there was a study on the influence of hematocrit on the result of the VerifyNow P2Y12 assay itself. Kakouros et al. identified a significant effect of hematocrit on VerifyNow P2Y12 assay after loading of clopidogrel, and they presumed that this effect was an in vitro phenomenon rather than a true physiologic action that is independent of intrinsic changes to platelet reactivity [31]. This inverse correlation between hematocrit and the PRU value of the VerifyNow assay has been explained as the difference in variance for blood sample turbidity resulting from ADP simulation, in which the changes in the optical signal transmitted blood samples with high hematocrit could not produce large percentile changes and would not translate the overall change of PRU value with high baseline [31]. Our result that increased hematocrit related to a lower risk of HTPR on prasugrel may be considered an issue of measurement methodology in the same context.

Despite HTPR being associated with periprocedural thromboembolic events [32], we did not find a significant association between HTPR and periprocedural thromboembolism. Periprocedural bleeding problems related to antiplatelet medications were absent, and there were eight cases of periprocedural thromboembolic complications. In these patients, only two were treated with the stent-assisted technique, while the others underwent simple coiling. None of the patients showed HTPR on prophylactic prasugrel, and in every case, coil loop protrusions into parent arteries were observed at the end of the procedure.

Our study has several limitations. First, this study was performed retrospectively, and the number of candidates was small. We did not find a significant association between HTPR or LTPR and periprocedural thromboembolic or hemorrhagic complications. Further research with a larger sample size and prospective study design is needed, in addition to studies investigating whether residual platelet reactivity after prophylactic loading of prasugrel, but also during maintenance of antiplatelet drugs, have significant associations with procedure-related ischemic or hemorrhagic complications in the neurointerventional field. A delicately tailored protocol for the dose of prasugrel premedication depends on the individual’s characteristics and must be addressed by further studies.

## Conclusion

In the endovascular treatment of intracranial aneurysms, high BMI and prior use of antithrombotic agents were related to HTPR of prasugrel premedication, and low BMI was associated with LTPR to prophylactic prasugrel. High hematocrit decreased the risk of HTPR on loading of prasugrel. Although we did not find a significant relationship between HTPR or LTPR on prasugrel and periprocedural thromboembolic or hemorrhagic events, respectively, when preparing the endovascular treatment for intracranial aneurysms, attention to patients with these clinical features is required to address the possibility of ischemic or bleeding complications.
